# Sustainable End-of-Life Management of Wind Turbine Blades: Overview of Current and Coming Solutions

**DOI:** 10.3390/ma14051124

**Published:** 2021-02-27

**Authors:** Leon Mishnaevsky

**Affiliations:** Department of Wind Energy, Technical University of Denmark, 4000 Roskilde, Denmark; lemi@dtu.dk

**Keywords:** wind energy, wind turbine blades, composites, maintenance, decommissioning, recycling, repair, biobased composites

## Abstract

Various scenarios of end-of-life management of wind turbine blades are reviewed. “Reactive” strategies, designed to deal with already available, ageing turbines, installed in the 2000s, are discussed, among them, maintenance and repair, reuse, refurbishment and recycling. The main results and challenges of “pro-active strategies”, designed to ensure recyclability of new generations of wind turbines, are discussed. Among the main directions, the wind turbine blades with thermoplastic and recyclable thermoset composite matrices, as well as wood, bamboo and natural fiber-based composites were reviewed. It is argued that repair and reuse of wind turbine blades, and extension of the blade life has currently a number of advantages over other approaches. While new recyclable materials have been tested in laboratories, or in some cases on small or medium blades, there are remaining technological challenges for their utilization in large wind turbine blades.

## 1. Introduction 

The development of wind energy is an important element of the strategy to limit global warming. A large expansion of the wind energy is expected in next decades. In Europe, 205 GW of wind energy capacity is currently available. A further 323 GW of wind energy will be installed in the EU by 2030 [1]. Wind energy now covers 15% of the EU’s electricity demand, and in 2030 it is expected to reach 30%. At the same time, a significant proportion of the installed wind turbines, the generation installed in the 2000s, will come to the end of their lifetime between 2020 and 2030 [2]. The share of installed wind turbines older than 15 years in Europe reached 28% in 2020 in general, and 41–57% in Germany, Spain and Denmark, in particular [3]. In 2021, a 4GW wind energy turbine (about 6000 turbines) can face decommissioning, due to the expiration of 20 years of support [4]. About 2.4% wind turbine blades have to be replaced per year [5,6]. In USA, about 8000 blades will be removed each year between 2021 and 2025. In Europe, 3800 are removed annually [7]. 

Many parts of wind turbines can be recycled; however, this is seldom the case for the composite wind blades [8,9,10,11]. The reason is that composite blades, which are the moving part subject to complex fatigue and environmental loads, are designed to sustain these loads for decades, and, thus, are extremely resistant to the loads also after the end of service time. Blades consists often of various materials elements, e.g., thermoplastic coatings, thermoset/glass fiber composites, often also carbon fibers, balsa wood, adhesives. All this makes it extremely difficult to separate the materials and recycle the parts. A total of 2.5 million tons of composite material are in use in the wind energy sector around the world [12]. More than 50 tons of plastic are contained in blades of a 5 MW wind turbine [13]. A total of 43 million tons of blade waste will be accumulated worldwide by 2050, 25% of them in Europe [14]. Each megawatt of installed capacity corresponds to 9.57 tons of composite waste (according to [15]), or even 12 to 15 tons (according to [16]). Annual composite wastes are expected to grow ~12% increase per year until around 2026, and then 41% per year until 2034, reaching 28,100 tons of blade material [6]. 

This paper reviews the various approaches and strategies of end-of-life management of wind turbine blades, from landfilling and incineration, via life extension, reuse and recycling, to the development of new smart, bio-based and biodegradable materials. 

## 2. Current Situation: Landfills and Incineration

**Figure 1:**
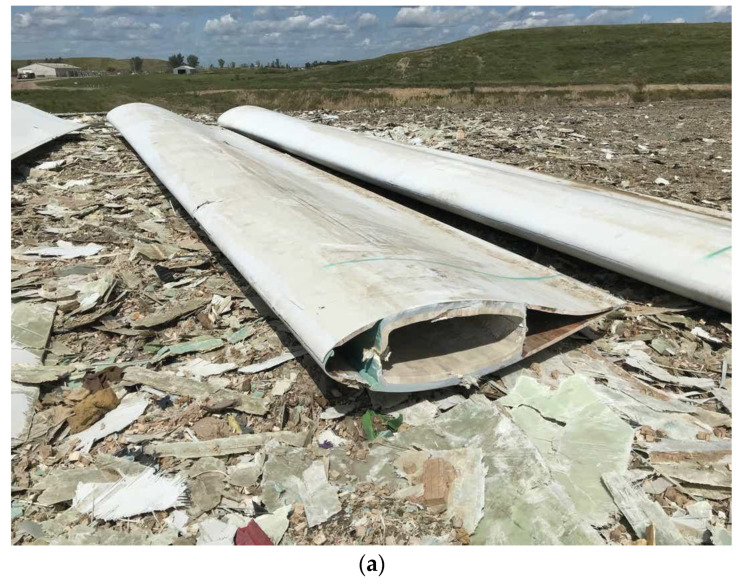

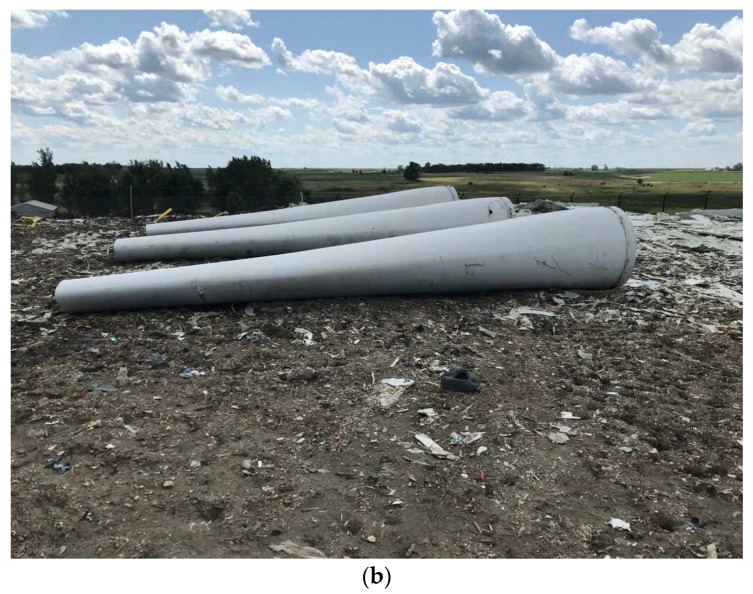
Old wind turbines in the in the Sioux Falls, USA, two views (**a**) and (**b**). The photo from [22] is reproduced with kind permission of Joe Sneve, Argus Leader.

The waste management hierarchy can be ranked from less favored to preferential option as follows: landfill energy recovery from wastes → recycling → reuse, → reducing the amount of waste [17,18,19]. In [20,21], the waste hierarchy for sustainable blade waste management is ranked (from less favored to preferential option) as: disposal → recovery → recycling → repurpose → reuse → prevention. Windmill blades can be landfilled in the USA [22], and are often incinerated in Europe [23]. Figure 1 shows old wind turbine, landfilled in the Sioux Falls, USA. 

The European Union issued a number of directives concerning composite waste control [17], including Waste Framework Directive (75/442/EEC) of 1975, amendment 91/156/EEC (which is an amendment of 75/442/EEC) and 91/689/EC (The Hazardous Waste Directive). The wastes are classified according to the European waste list 2000/532/EC. The EC Incineration Directive (2000/76/EC) sets requirements for waste incineration plants. The EC Landfill Directive (1999/31/EC) deals with waste minimization and recovery, including recycling. The European Waste Framework Directive (2008/98/EC) defines the concepts and definitions related to waste management. Still, when blade decommission is a part of life cycle assessment (LCA), the majority of studies assumes either landfilling or incineration of used wind blades in their analyses [24]. Most LCA do not include end of life management of blades at all.

## 3. Reducing the Amount of Wastes: Service Time Extension

**Figure 2:**
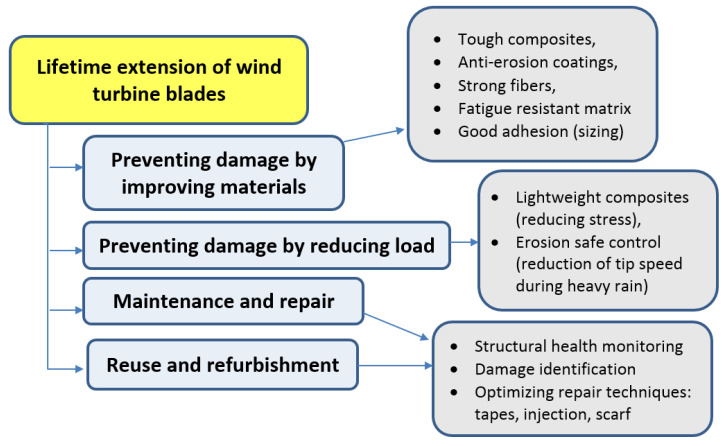
Schema: Extension of lifetime of wind turbine blades: Approaches.

One of the approaches is to delay the problem, by extending the durability of the blades. As noted in [25], durability is one of the “most obvious strategies for reducing waste and increasing material productivity”. There exist several options to increase the service time of wind turbine blades, among them, using more durable materials, ensure better maintenance, repair, reuse and refurbishment [26]. Figure 2 shows a schema of various approaches of the extension of lifetime of wind turbine blades. The different ways of lifetime extension of blades are discussed in more details in the following sections. 

### 3.1. Durability of Blades: New Materials Extending the Blade Lifetime

Razdan and Garrett [27] calculated global warming potential (GWP) of Vestas onshore V112-3.45 MW turbines as a function of the lifetime. With increasing the lifetime from 16 to 20 and 24 years, the GWP decreases from 6.6 g CO_2_-e per kWh to 5.3 and then to 4.4. Jensen [28,29] made an assessment of the environmental impact on extending the lifetime, based on a life cycle assessment for a 3.2 MW onshore wind turbine. According to this estimation, the kg CO_2_ /MWh was 8.7 for 10 years lifetime, 4.4 for 20 years and 2.8 for 30 years. Thus, the extended lifetime has a clear positive impact on the carbon footprint. The lifetime of wind turbine blades is controlled by the properties of the blade materials and the applied loading. The extension of planned lifetime can be based on understanding the degradation mechanisms of blades, using better materials, reduced loading and changed design or, in some cases, optimal maintenance strategy [30]. 

Damage mechanisms of wind turbine blades include surface erosion, debonding and adhesive joint degradation (skin/adhesive debonding, adhesive joint failure, sandwich debonding, delamination, splitting along fibers, cracks in gelcoat) [31,32,33,34,35]. Several strategies are used to increase blade durability and prevent the degradation. In order to prevent surface erosion, new engineered coating materials are developed [36,37]. The approaches to develop durable composite laminates include using lighter and stronger fibers (for instance, high strength glass fibers with modified compositions, like S-glass, R-glass, carbon, basalt fibers) [30], hybrid composites [38,39], using special fiber sizing and nanomodification of matrix, to increase fatigue resistance of polymer matrix [40,41]. The use of lighter materials (for instance, carbon fibers versus glass fibers) allows reducing the blade weight, and, thus, ensure lower weight loads on the blades. For erosion protection, a load reducing strategy can be realized as so-called erosion safe control, i.e., reduction of tip speed during heavy rain [42,43]. 

While different materials ensure different durability of blades, they have also different climate effects: manufacturing of carbon fibers causes 100 kg CO_2_/kg, while this parameter is far below 20 for epoxy and glass fibers [44].

Recently, one more approach to the extension of durability of wind turbine blades attracted growing interest, namely, easy-healing or self-healing materials [45,46,47,48]. Several technologies for the development of self-healing or easy-healing lightweight structural composites are under development now, with different degrees of usability: capsulation and microcapsulation, hollow fibers, vascular networks, also supramolecular polymers, vitrimers, Diels-Adler healable polymers. In all the cases, only polymer matrix, adhesive bonds can be made self-healing. The self-healing mechanisms are potentially usable only for non-structural damage. The majority of self-healing solutions rely on external stimuli (like heating). Several self-healing solutions involve the incorporation of self-healing agents within a brittle capsule or vessel into the polymeric matrix [46]. Microencapsulated systems are based on an epoxy precursor, a micro-encapsulated healing agent and a catalytic chemical trigger within epoxy matrix [47]. While micro-encapsulation allows autonomous activation, it also increases manufacturing complexity and leads to reduced mechanical performance. Vascular networks [48] also can reduce the mechanical performances of the blades. Arun Kumar et al. [49] implemented the vascular channel self-healing in wind turbine blades. Still, 25% decrease in tensile strength and 9% decrease in the flexural strength were observed with the inclusion of a single layer of vascular vessels in the composite. Diels-Adler self-healing technology [50] allows repeatable healing, however, the material is typically too soft for structural applications. Vitrimers show relatively high mechanical strength, are insoluble and still heat processable, and recyclable. 

The application of Diels-Adler self-healing and vitrimers is related to the reversible polymers, which have a potential not just “heal” defects, and extend the lifetime, but also open the possibility of full recycling of blades. These materials are discussed below, in Section 5. The self-healing composites represent an interesting solution for the extension of the blade lifetime, and, thus, reducing the wastes. Most of the available solutions are now still at the lower or medium technical readiness level (TRL). 

One should be noted that including more different material in the blade like carbon fiber and glass fiber conflicts with circular design of composites and recycling at end of life. Less combination of materials and broader using of the same materials would be make blades more sustainable. This idea was realized in the self-reinforced polymers, see [51].

### 3.2. Failure Control and Correction: Maintenance and Repair

The strategies discussed in the previous section (development of new extra-durable wind blades, designed to survive additional years or decades of service) represent “proactive” approaches, which can be implemented only in following years or decades, when new wind turbines are installed. The reactive options to increase the service time of blades (already installed) mean improved maintenance and repair. This includes health monitoring, damage reporting and identification (using the visual inspection, observing the material property changes or non-destructive inspection techniques), repair procedures, and quality control. 

Maintenance of wind turbines is an expensive part of the wind turbine functioning, taking in average up to 20–25% of the total levelized cost per kWh over the lifetime of a turbine [52]. The maintenance can be realized as corrective or preventive strategy. The corrective maintenance is initiated, after the failure or damage event takes place, is noticed, and reported. Due to rather late start of maintenance activities, corrective maintenance strategy can lead to large failure of wind turbines. The preventive maintenance can be realized as scheduled (regular inspections of wind turbines) or as condition-based maintenance. As intermediate solutions, reliability- and risk-based inspection strategy are employed, when the next inspection is scheduled on the basis of reliability prognosis and crack growth analysis, or with view on the consequence of a failure and inspection versus repair costs [53]. Condition based maintenance requires the permanent health monitoring of the wind turbine. In order to realize the structural health monitoring, wind turbine blades should be modified, for instance, by attaching permanent sensors to the blades. Different technologies of structural health monitoring are described in details in [54,55,56,57,58]. The condition-based maintenance is very promising approach, and some solutions already exist on the market, e.g., Gram and Juhl developed system based on vibration sensors, or optical sensors [59]. 

After the damage in the blade is identified, various repair techniques can be employed: coatings, tapes, shields (for surface erosion repair and protection), filling and sealing, resin injection (for small surface cracks or small delaminations, other non-structural matrix cracks) and plug/patch, scarf repair (structural damage, including the cracked fibers) [32]. On average, a new repair is required after 1 to 3 years, and the increase of after-repair (or between repair) time reduces the energy costs. 

**Figure 3:**
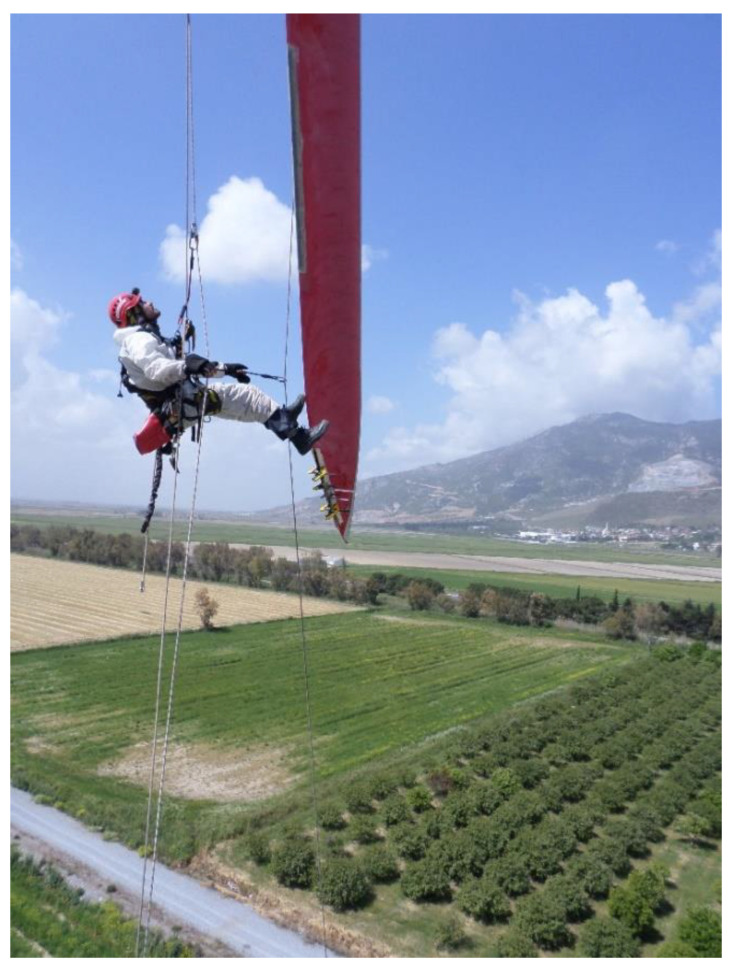
On-site repair of wind turbine blade. Reprinted with kind permission from Mira Rope Access, http://www.mira-ra.com, (accessed on 13 January 2021).

**Figure 4:**
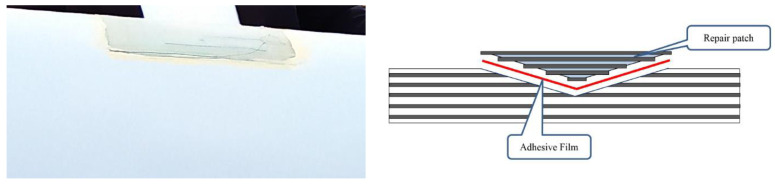
Repaired leading edge of the wind turbine blade (**left**) and computational model of scarf repair developed (**right**) in [62]. Reprinted with kind permission of Springer.

Structural repair is carried out by attaching the repair patch/scarf to the parent laminate [32]. The repair can be carried on site (which can take ~21 h of technicians work and cost of the order of 4800 EUR [31]), or transported to the factory (which costs much more). Figure 3 shows a photo of on-site repair of wind turbine. The patches can be applied as composite patch co-cured with the adhesive (soft patch), pre-manufactured patch in mold (hard patch molded), machined hard patch, and semi-hard patch (series of pre-cured composite laminates, each with several plies, interleaved with adhesives and bonded) [60], as pre-cured thin patches, with glue between them [61]. Figure 4 shows photo of a wind turbine blade with patch, and a computational model of a coating/patch/adhesive/parent structure system, developed in [62]. 

In a number of technologies, various heating or radiation effects are used to control the curing process during the repair, allowing both shortening the repair time and better repair quality, e.g., ultraviolet light (UV) quick curing technology by Gurit [63], laser heating for better bonding [64]. 

Improving the quality of maintenance and repair and reliability of repaired blades is an important element in the extension of lifetime of the wind turbine generation established in the 2000s and earlier. Repair technology is also a necessary part for the refurbishment and reuse of the wind blades. 

## 4. Recycling of Wind Turbine Blade 

While the life extension and re-use efforts can delay the end-of-life of the blades, at some stage, old blade have to be incinerated or recycled. Different recycling technologies for currently used thermoset composites have been developed. The recycling methods (for plastics and composites) are classified into following groups [65]: primary recycling or reuse (“recycling products for the same use”), secondary recycling (“recycling products for uses other than their original use”), tertiary recycling (“recovering petrochemical components of plastics via a chemical process”), and quaternary recycling (“incinerating plastics to recover energy in the form of heat”). 

In [66], the recycling technologies are classified into “first period” technology (1980 to mid-2000, with the goal to recover fibers), “second period” (incorporating recovered fibers in new resin, 2009–2012), and “third period” technology (“direct structural recovery”, maintaining the original resin/fiber structure instead of separating composites into recycled fiber and recycled resin). In the “first recycling period”, the mechanical (shredding, cutting), and thermal recycling technologies (combustion, pyrolysis, and fluidized bed) have been developed. The “first recycling period” technologies include the so-called secondary and tertiary recycling, based on mechanical, and chemical or thermal processes, respectively [67,68,69]. The constraints of these techniques are, generally speaking, that the polymer matrix is simply destroyed, while the fibers are often damaged and still their remains (e.g., short fibers) can be covered by polymer remains, thus, making them to lower quality reinforcement in the composites. In the “second period”, the research for enhancing the recovered fibers for the further use in new composites via modified fiber surface properties were carried out. In particular, the thermal and chemical or solvolysis-based recycling are modified, and surface reactivation is used to improve the finer adhesion. This allowed making the output fibers to be a better quality reinforcement for recycled composites. The research in the “third period”, according to [66], are devoted, among others, to thermoforming the composites into new products using catalysts for reversible crosslinking chemistries and reintroducing into resin as reinforcement into new products. The reversible chemistry and other low TRL technologies have apparently limited applications to the wind turbines installed in the 2000s, and will be discussed in the next section. Detailed reviews of various recycling technologies are given in [70,71,72,73,74,75,76].

### 4.1. Primary Recycling: Re-Use and Refurbishment

Before involving the composite materials recycling technologies, the possibility of re-use of wind turbine blades should be considered, if the remaining technical resource of a blade is still available. This can be the case if blades are replaced due to economic reasons (e.g., subsidy period). The authors of [26] proposed to extend lifetime of wind turbines using the safety evaluation. The procedure includes analytical evaluation (comparison of operating loads with design loads), verification of structural stability (load bearing and safety parts, calculation of potential duration of continued operation) and on-site inspection, to document damage or unusual wear and tear. Refurbishment of wind turbine blades can allow enlarging blade size, improving structural parameters, removing defects [77,78]. For instance, around 20% of the blades in the Netherlands are refurbished and resold to buyers within and outside the EU [79]. 

Several companies offer turbine upgrading approaching, among them, SGRE’ Life Extension Program, extending the useful life of wind turbines by ten years, Vestas PowerPlus^®^, increasing the wind energy production by up to 5% annually, and General Electric platform Wind PowerUp, which also allows 5% increasing the wind farm’s output and 20% higher profit per turbine [80]. In 2013, Vestas launched the Wind for Prosperity program, to deploy green technology in third world countries, by factory-refurbish a selection of wind turbines that have favorable dimensions for transportation and erection.

**Figure 5:**
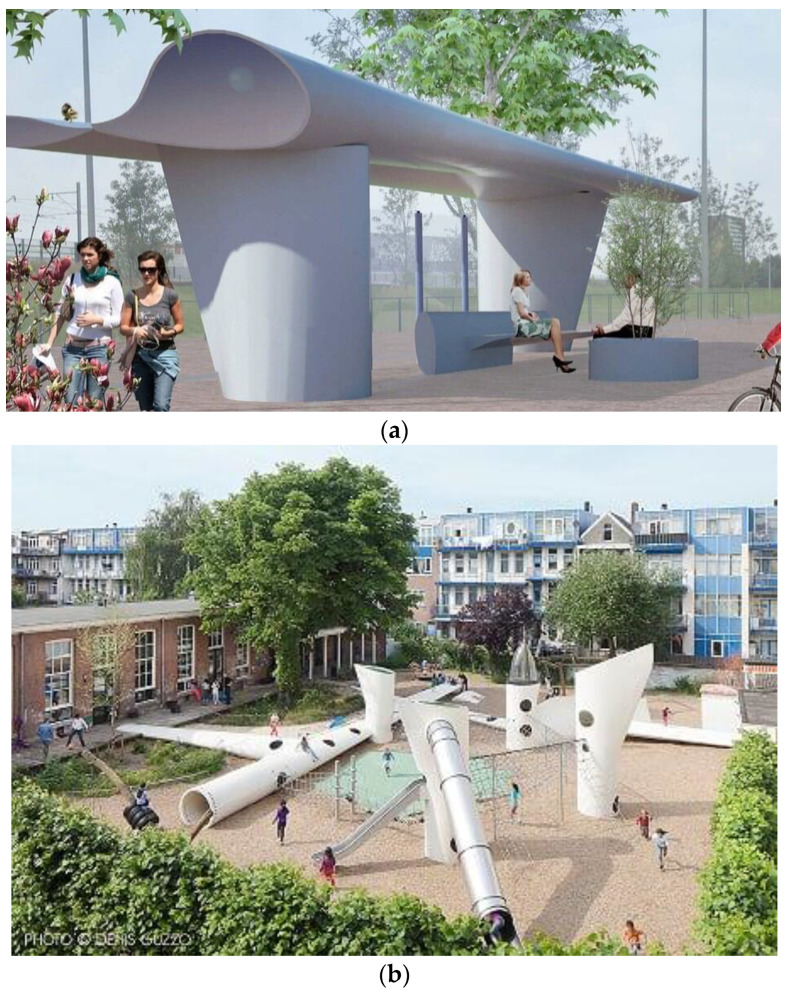
Re-use of wind turbine blades, in station (**a**) and in playground (**b**). The photo of the design by Superuse Studios is reproduced here with kind permission of New Citizen Design and Dennis Gusto Photography.

An alternative to the re-use of wind turbine blades for their direct destination is the re-use of blades or blade parts in different structures. The Dutch firm Superuse Studios proposed using blades in architecture, as bus shelter, city benches or playgrounds [81]. Figure 5 shows an example of these structures. Other applications include bridges (which requires additional testing and special design efforts), playgrounds, bus shelters, and other urban furniture [70,82,83,84]. Bank et al. [85] suggested to re-use parts of wind blades in new or retrofitted housing projects in Mexico, where harsh environmental conditions (water and high humidity) exist. The 100 m long blade was used for foundation, roof frames and interlocking roof systems of homes [85]. 

### 4.2. Secondary (Mechanical) Recycling

The secondary type of recycling involves mechanical modification of the materials, without the use of chemical processes, e.g., breaking the composite by shredding, crushing, milling, mechanical separating into resin and fibrous products. Polymer composites remain strong materials even after the twenty years long service, with some defects. By employing mechanical disintegration, the composite is separated into smaller but still strong parts, which can be then used as reinforcements in various products. In [86], high voltage fragmentation was investigated as a potential process for composite recycling. 

**Figure 6:**
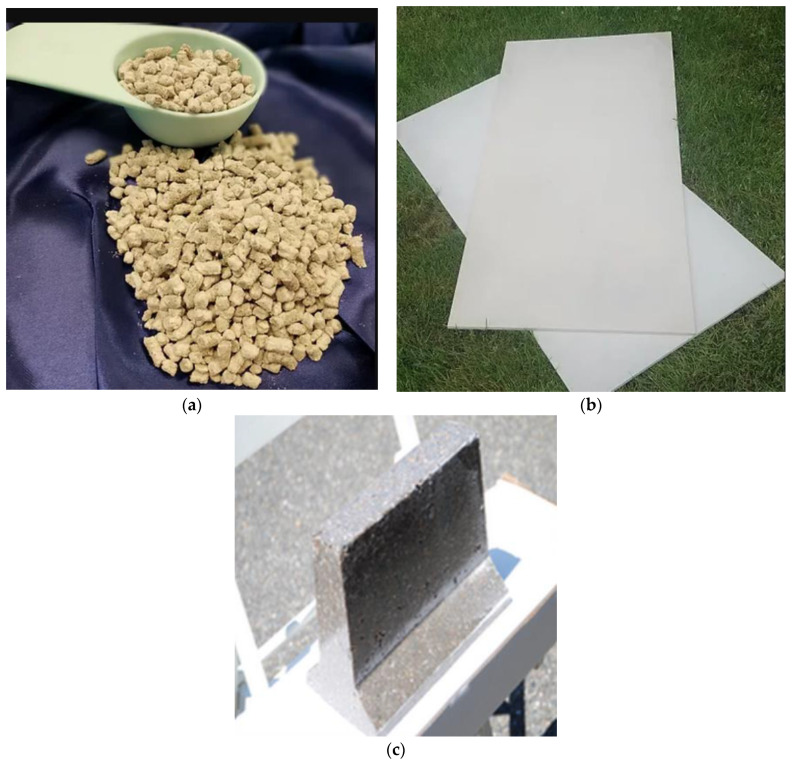
Products Global Fiberglass Solutions made from fiberglass wastes: EcoPoly Pellets (**a**), EcoPoly Panels (**b**), Road way Applications (**c**). Photos are provided by Global Fiberglass Solutions.

US company Global Fiberglass Solutions developed a technology to break down blades and press the parts into pellets and fiber boards, which are used in flooring and walls. Figure 6 shows the products, made by Global Fiberglass Solutions from fiberglass wastes, among them EcoPoly pellets, EcoPoly panels, and road way applications. 

Cement producer LafargeHolcim, via its business unit Geocycle, developed technology to use crushed blade dust in cement production plants [87,88]. The blades are cut, shredded in smaller pieces, and the crushed blade dust is mixed with a humid material, to homogenize and bind together the dust, and the end product is ground into a powder creating cement. This solution is cheap and can be used for many tons of blades. Additionally, Geocycle used the resin matrix as an alternative fuel, substituting other fuels. In the EU FP5 Growth REACT project, composite materials were disintegrated through pure mechanical granulation or pyrolytic cracking, and the plastic surrounding reinforcing fibers were separated and used as active filler material. Beauson et al. [89] manufactured and tested polyester resin composites reinforced with shredded composites from wind turbine blades. In the tests, low failure strain values, and bad adhesion of the shredded regions with the matrix were observed. Generally, the mechanical composite destruction (shredding, crushing, milling) represents only one intermediate step in the recycling technology, and is followed by thermal or other steps, to separate materials parts. 

### 4.3. Tertiary Recycling (Recovery) of Wind Turbine Blades

The tertiary recycling of composites is carried out by thermal (pyrolysis), or chemical decomposition (solvolysis). Pyrolysis process is carried out by heating the material between 450 and 700 °C in the absence of oxygen [76]. In this way, the polymer matrix is converted into a gas, oil and char, while the fibers remain inert and can be later recovered. The material is downsized before pyrolysis. 

**Figure 7:**
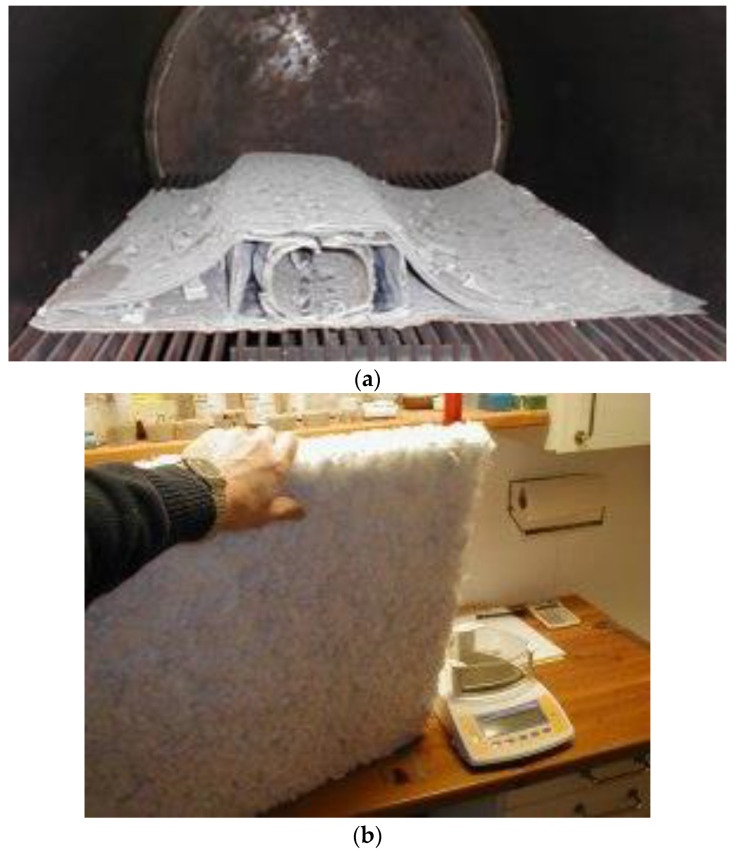
Wind Turbine Blade part after pyrolysis and Insulation (**a**) and Wool Mat made from blade fibers (**b**). Photos are provided by ReFiber ApS and Erik Grove-Nielsen, Nordisk AeroForm ApS.

In Danish company Refiber ApS, the ReFiber process was developed, in which the plastic part is gasified in an anaerobic atmosphere in a rotary kiln (pyrolysis chamber), and then the glass fibers are recovered, separated and cleaned. The resulting fibers have 50% reduced strength, while the stiffness of the fibers is unaffected by the thermal treatment. They can be used as insulation materials or as fiber reinforcement. Figure 7 shows the blade part after pyrolysis and wool mat made from the fibers.

In the project FiberEUse [90], Tecnalia developed thermal treatment based on a low temperature oxygen-free pyrolysis process, which allows it to partially retain the strength of the glass and carbon fibers. The purpose was to recover both fibers, as a whole fabric, to be reprocessed by resin transfer molding technology, or alternatively, preparing fibers for compounding. The fibers are then re-used in automotive components, or building components like roof light panels and valley gutters for roofs. 

In the fluidized bed pyrolysis, a “bed”, fluidized by hot air is used, which allows quick heating of the materials [90,91]. Oxidation in the fluidized bed is carried out by combusting the polymeric matrix in a hot and oxygen-rich air flow of 450 to 550 °C [92,93]. The fibers are then contained in the air flow, and can be separated. The pyrolysis processes can involve microwave heating for polymer degradation [94,95]. Åkesson et al. [94] studied microwave pyrolysis of wind turbine blades, and observed that glass fibers lost about 25% of their strength.

In solvolysis, the chemical decomposition of the epoxy matrix is carried out using reactive solvents, for instance, nitric acid, ammonia or glycol, below critical temperature ~100 °C, or water or ethanol, near at critical temperature [70]. As a result, pure fibers without resin, and decomposition materials are available. In [96], solvolytical conversion of the polyester matrix was proposed; this approach involves producing potentially valuable oil fuel with heating values up to 39.6 MJ/kg, simultaneously with the recovery of glass fibers. 

In chemical recycling, special solutions (catalytic, benzyl alcohol or supercritical fluids [67,97,98,99]) are used to decompose the polymer, while fibers retain most of their properties. Benzyl alcohol and water in subcritical and supercritical conditions were used to recover carbon fibers from composite materials in [100], ensuring complete separation of the composites and clean recovered fibers. Chemical recycling is one of the most promising recycling methods due to more clean and higher strength fibers and due to lower temperatures vs pyrolysis. 

Mattson et al. [101] described challenges of chemical recycling of wind turbine blade composites, using solvolysis/HTL (hydrothermal liquefaction) methods with subcritical water as solvent. They noted the multiple materials of blades and necessity of their separation as additional challenge of the technology (comparing to the pure thermoset based composites). A multitude of investigations have been published during the years regarding solvolysis of newly produced composite laminates and known thermoset composition (epoxy, polyester, and vinyl ester).

In several works, the surface properties of recycled fibers were enhanced, by obtaining cleaner or active surfaces [66]. This can be achieved by changing thermal regimes, e.g., modified pyrolysis methods using lower temperatures for carbon fibers [102], or higher temperature for thermolysis to achieve low char residue on recovered fiber [103], or using additional chemicals to resize recovered fibers or remove residues [104,105,106]. Another direction is the manufacturing of thermoset flakes from blade materials, which are then incorporated in thermoplastic resins [66,107], or epoxy resin [108], and then molded or thermoformed into sheet products. The output materials show better properties, than other recycled materials.

The recycled fibers or crushed composites can be used in various secondary applications, e.g., in concrete production, also with micro-silica additions, particleboard, wall paints [70], precast concrete elements, or internal cores in multilayer panels with recovered fibers [109]. The developed recycling solutions have some constraints: low homogeneity and high variability of the mechanical recycling products, low quality of produced fibers, shorter recycled fibers, energy consuming pre- and post-processing stages, variability of blade designs and materials, high processing price [70]. According to Jensen and Skelton [70], all the blade recycling processes are lacking business case, with relatively expensive recycling operations and the lack of a market for the recycled materials.

## 5. Preventing Composite Wastes-1: Recyclable Polymers

Re-use, recycling, recovery and remanufacturing of wind turbine blades, now coming to the end of their life, represent a challenge for the energy industry, causing additional costs. Now, in the 2020s, new wind turbines are developed, manufactured and installed. With view on the end-of-life of new and future wind turbines, the problem should be solved in its roots, by developing sustainable, recyclable wind turbine blades, to prevent the reappearance of these problems in future. The works to develop sustainable, recyclable wind turbine blades are carried out in several directions, among them, recyclable strong polymers, thermoplastic polymers, bio-based and biodegradable materials. 

The composites are made from strong, stiff fibers, and a tough polymer matrix. The first approach to make composites recyclable involves making the polymer matrix easily removable, degradable or even reusable. This allows also reuse of the fibers. This can be done by using thermoplastics matrices, instead of thermosets, or by using recyclable thermosets, instead of common epoxy or polyesters. This option is considered in this section. According to [44], sufficient reduction of lifetime emission can be achieved by recyclable resin system: for instance, for 8MW turbine, the lifetime emission reduction reaches 28%. 

**Figure 8:**
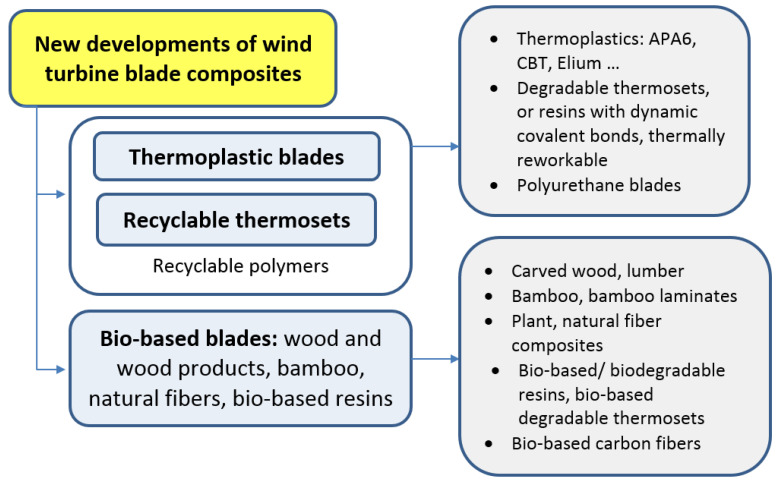
Schema: New directions of the development of wind turbine blade materials.

An alternative way is to use bio-based or/and biodegradable fibers or lumbers (in best case, combined with bio-based and biodegradable matrix), and is considered in the next section. Figure 8 shows a schema of new directions of the development of wind turbine blade materials. 

### 5.1. Thermoplastics Composites for Wind Turbine Blades

The advantages of thermoplastic resins include the shorter manufacturing cycle, possibility of joining without additional adhesives, and recyclability by heating. The disadvantages include high temperature processing, drying and cooling requirements, surface treatment requirements. In a number of works and projects, the possibility to develop wind turbine blades from thermoplastic polymers has been explored [110,111]. Lystrup [112] explored the potential of polypropylene and polyethylene polymers, studied the fatigue performance of glass fibers with these systems. Tusavul et al. [113] investigated the injection molded thermoplastic polyether ether ketone (PEEK) with 40% carbon fillers, as a replacement for thermoset composites, and demonstrated that the material can potentially provide up to 28% reduction in weight and higher stiffness without significant reduction in elongation.

In the Duwind project (TU Delft), a technology of vacuum infusion of thermoplastic composites was developed [110,114,115]. The thermoplastic polymer used was anionic polyamide-6 (APA-6), with viscosity one tenth of that of epoxy and low processing temperature. Van Rijswijk applied this technology to manufacture 25 mm thick thermoplastic composites with a fiber volume content of 50%. Joncas [116] analyzed the usability of reactively processed APA-6 in blades and proposed special topology-optimized blade design. He demonstrated also recycling of APA-6/glass long-fiber composites into APA-6 short-fiber reinforced composites by regrinding and injection molding, with rather good properties.

Irish company ÉireComposites manufactured 12.6 m long blade using cyclic butylene terephthalate (CBT), using the own heated ceramic/thermoplastic tooling technology.

Garate et al. [117] developed vacuum-assisted thermoforming for manufacturing of small segments of thermoplastic (polypropylene resin) composites, and tested the segmented wind blades at a small-scale wind farm. In the Danish BladeKing project, the group of universities and companies sought to develop an innovative technology for manufacturing wind turbine blades, also considering thermoplastic polymers. Durai Prabhakaran [118,119,120] reviewed applicability of commingled (hybrid yarns), prepreg (pre-impregnated tapes), or reactive based polymers (anionic polyamide 6/APA6 and cyclic butylene terephthalate/CBT) for wind turbine blades. Durai Prabhakaran concluded that the reactive polymers have potential to replace the thermoset resins in large blades, thanks to their low viscosities. Durai Prabhakaran et al. [118] also compared mechanical properties for various glass reinforced thermoplastic laminates (flexible, compression strength, moduli, interlaminar shear strength). An important subject is the development of fiber sizing, which should prevent the properties (in particular, interface properties) decrease due to moisture uptake [110]. The authors listed the main challenges of application of thermoplastic composites in wind blades, among them, high temperature processing and thus higher blade costs, drying requirements, faster cooling rate to minimize void formation, fiber adhesion and surface treatment requirements, control of resin flow. Extensive research on fiber surface treatments and sizing is also underway to augment the fiber/matrix chemical bond, further increasing static and fatigue properties.

**Figure 9:**
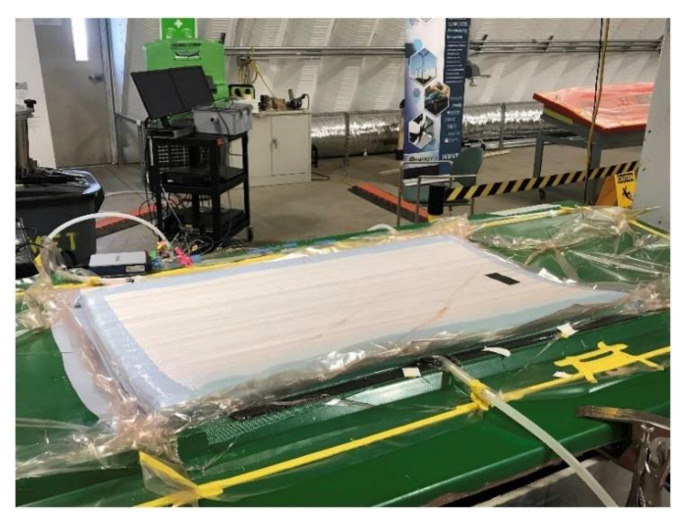
Elium spar cap component fabricated at the National Wind Technology Center. Reproduced from [124] with kind permission from Elsevier.

French company Arkema developed Elium^®^ liquid thermoplastic resin for the manufacture of composite parts, with the same technologies and processing equipment, as for thermoset composites (see Figure 9). In the French Effiwind project, the partners sought to develop and validate technologies of manufacturing Elium blades and produced the first 25-meter blade in 2016. Elium shows the mechanical properties, close to those of epoxies, but much higher toughness. 

A group at National Renewable Energy Laboratory (NREL) studied the manufacturing process for specific parts of a thermoplastic wind blade, including thermal welding and fusion joining, and manufactured a 13-meter thermoplastic blade [121]. The authors demonstrated also that fusion joining can replace adhesives, ensuring an increase in both the static and fatigue strength as compared to bonded coupons [122]. Murray and colleagues [123] developed manufacturing processes of thick glass fiber-reinforced acrylic thermoplastic resin wind turbine blade spar cap. They concluded that the thermoplastic resin system can be used for the manufacturing of wind turbine blades using vacuum-assisted resin transfer molding. They also demonstrated the recycling of thermoplastic wind blade by dissolution, so that the recycled materials show the same mechanical properties as virgin materials [124].

There exist still challenges in using thermoplastic polymers in large wind blades, among them, moisture sensitivity and requirement of drying cycles, low cooling rate in thick laminates, heterogeneity of materials properties due to different cooling rates, and narrow processing window. Still, recent developments provide solutions to some of these problems. 

### 5.2. Recyclable Thermosets

Mechanical properties and performance of recyclable thermoplastic-based composites, discussed above, and also of bio-based composites, are still far behind of those of currently used, common thermoset based composites, e.g., glass/epoxy. Therefore, the idea arose to develop composites with recyclable thermosets, thus, combining the best of two worlds: recyclability and high performance of cross-linked thermosets. 

Thermoset recyclability can be ensured by introducing degradable or dynamic covalent bonds in the polymers. The dynamic response of these bonds should be triggered by an external stimuli [125]. The external triggers can be, for instance, thermal or acidic loading, catalysts or ultraviolet light induced stimuli. Garcia and colleagues [126] reported the development of recyclable thermosets which can be returned to their monomeric state using a pH trigger [127]. 

Pastine and colleagues [128,129] developed recyclamine hardener for epoxy resins that enables recycling of thermoset composites. The breakage of thermoset cross-links can be achieved by thermal and pH load. 

The extent of the material degradation differs, depending on the degradation mechanism, and can destroy the polymer into non-reusable (e.g., gases) or reusable systems [125]. Epoxy can be designed for the decomposition with external chemical triggers (like acetals in tetrahydrofuran and hydrochloric acid, esters in aqueous sodium hydroxide, and aminals in concentrated sulfonic acid, see [130]); however, it would require large scale application of organic solvents. Development of disulfide-containing hardeners will allow cleavage of hardener molecules and thus, degradation of the epoxy [130,131]. Wu et al. [132] developed a recyclable epoxy resin based on a cleavable curing agent which can be chemically separated from fibers and recycled under moderate conditions. In [133], a recyclable carbon fiber composite, with degradable thermosetting poly(hexahydrotriazine) resin, which allows multiple intact recoveries of fibers and near-total recycling of the matrix through gentle depolymerization in dilute acid solution, was developed. 

The triggered degradation of thermosets can be realized by cleavable linkages in the polymer network. In this way, the energy and temperatures required to degrade the polymers are lower, at the level of 200 up to 300 °C, but still do not cause material degradation during service time. Such thermosets are called thermally reworkable [134]. There are a number of requirements to the degradation stimuli: they should not trigger degradation during service time or reduce performance (like water or salt), but also the environmental effect of used solvents (which can be then potentially applied to many tons of polymers, if the development is successful) should not have negative environmental effects. 

Bio-based thermosets, that degrade in specific biological media, are currently under development [125,135]. In the Danish project DreamWind (Designing Recyclable Advanced Materials for WIND energy), production of stimuli-responsive materials, which can be disassembled after use, relies also on incorporating bio-based resources in the new materials. It is of interest to mention here also the 55.2-meter-long wind turbine blades, based on a thermoset polyurethane infusion resin solution [136], developed and installed by the company Covestro. This opens new possibilities for recycling. 

The recycling of thermosets, maintaining the overall polymer structure, can be realized using the dynamic covalent chemistry technology, discussed also above (as a way to enhance the blade lifetime by self-repair) [137]. The materials, potentially usable as self-repairing composites extending the service life, provide also the option of easier and more efficient recycling. There are several works where structural composites with vitrimer matrix were developed [138,139]. Schmidt and Reynaud and colleagues [140,141,142] achieved reworkability of their bio-based epoxy/glass fiber composites through the introduction of transesterification catalysts.

Generally, reversible high performance thermoset based composites represent a promising direction for the recyclable structural composites; however, they are still in development stage, with relatively low technology readiness levels. There are still many development stages before the materials and related technologies can be considered for wind turbine blades. 

## 6. Preventing Composite Wastes-2: Bio-Based Composites

### 6.1. Wood and Wood Products

Wood was the material used in ancient wind turbines in Egypt and Persia [143]. Wood is the widely available and clearly sustainable material, and has high stiffness to density ratio and high fatigue resistance. Thus, it is obvious to employ wood, or wood-based products for manufacturing the wind turbine blades. In the early 1980s, renewable materials such as birch, Douglas fir and spruce, as well as bamboo, were being considered for large horizontal axis wind turbines in the US [144,145,146]. Birch has proven to be very successful as a primary material in large horizontal-axis wind turbine blades. 

Various possibilities of using wood elements in structures are available, e.g., solid wood, glue laminated timber, laminated veneer lumber, wood strips and veneers.

**Figure 10:**
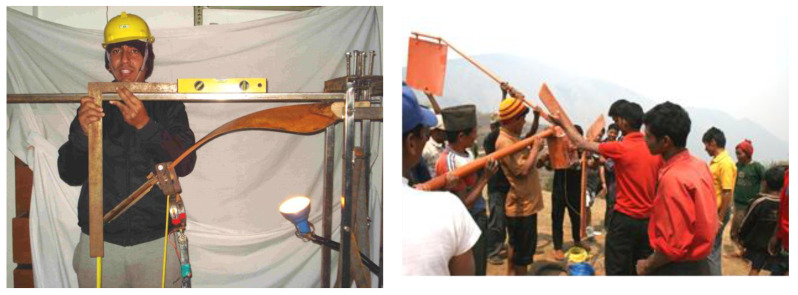

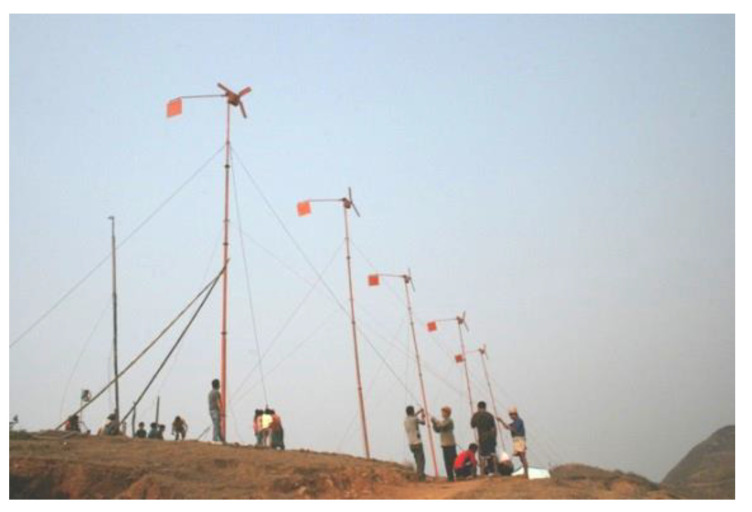
Locally produced wooden wind turbine in Nepal: testing and installation in Dhulikhel region. The figures are reprinted from [147] with kind permission of Elsevier.

In the simplest version, the blades can be carved from wooden parts, fully or in parts. Mishnaevsky Jr., Sinha et al. [147,148] demonstrated the applicability of Nepali timber for low cost wind blades. The turbines with timber wind blades were installed on several locations around Nepal, and their usability was demonstrated (see Figure 10). Astle et al. [149] studied material and fatigue properties of Douglas fir and Sitka spruce and the manufacturing routes for small wind turbine blades, also using copying route. Pourrajabian et al. [150] tested different species of timber for use in small blades. Design and optimization of solid and hollow blades for a small horizontal axis turbine via genetic algorithms are presented. However, the technology of solid wooden blades can be used only for small wind turbines. Other approaches to wooden blades include wood construction (with a framework of ribs and stringers covered by planks) [151,152], wood epoxy blades (wet layup of impregnated wood veneers) (like that developed by Gougeon Brothers [153]), blade shells produced by vacuum infusion of wood with pultruded strips of carbon fibers (developed by NEG Micon). 

The most common application of wood based composites is in construction industry, in the form of structural composite lumber (SCL), which are available in the form of laminated veneer lumber, parallel strand lumber (veneers chopped into long strands, pressed and laminated into beams), laminated strand lumber (shorter strands, oriented and laminated into beams). Laminated veneer lumbers have more uniform and predictable properties than solid wood parts [154]. Borrmann [151] also suggested a new blade concept, based on the laminated veneer lumber (LVL) combined with carbon fiber reinforced spar cap. The blade manufacturing in this case is realized not via negative molding but by numerically controlled milling. The blade is designed from laminated veneer lumber with unidirectional and bidirectional layers, and UD carbon fiber reinforced plastics. Borrmann demonstrated that it is possible to design a wood-CFRP wind turbine blade for the NREL 5 MW wind turbine under onshore conditions according to IEC 61400-1. According to [155], wood-flax hybrid blade (wood skin, root, shear webs, and trailing edge with a carbon spar cap) is most competitive of the full bio-based designs, compared to all-flax and all-wood. 

In 2015, a 5 MW wind turbine with 61.5 m long blades, was constructed at the University of Massachussets at Amherst. It was observed that a blade made of laminated wood veneer panels would be 2.8 heavier than a plastic blade (48 versus 17 tons) and have a laminate of over 50 cm thick [155]. Although this suggests that it is technically possible to build a wooden blade more than 60 m long, it is not very practical. With heavier blades, the wind turbine needs to be built much stronger, which increases the costs and the use of resources. The major disadvantages of the wooden rotor blades are difficulties to obtain the chosen wood products in high and reproducible quality, as well lower stiffness of wooden composites (thus, requiring glass or carbon elements). 

One area where laminated wood products can be well used is turbine towers. The German company TimberTower built a 100 m tower from laminated wood in 2012. The Swedish company Modvion built a 30-meter-high tower from cross laminated timber, and works on a 110- and even a 150-meter-high tower.

Generally, it is observed that the wood or laminated wood products, while tough and fatigue resistant, demonstrate still inferior mechanical properties as compared with the glass epoxy composites. These materials can be used in combination with much more stiff carbon fibers. Carbon fibers are manufactured typically from polyacrylonitrile (PAN). In a series of projects [156,157], alternative environmentally friendly processing routes with bio-based precursors are developed, which allow potentially replace the common carbon fibers by bio-based fibers. Currently, these fibers show still (again) inferior stiffness and strength as compared to the usual carbon fibers. 

There exist a number of challenges with application of wood or laminated wood products for wind turbine blades, among them, moisture sensitivity (which still can be solved by choosing special coatings), variability of structures and properties (which can be potentially solved by using standard rules for wood source choice, veneer technology, manufacturing), still relatively low stiffness/weight of wood as compared to glass fiber/epoxy (can be solved by combining wooden and carbon fibers elements). A number of projects in this area are currently underway. 

### 6.2. Bamboo Based Composites 

Another quite promising natural material for structural applications is bamboo. Bamboo has high specific strength and modulus, and high fracture toughness (as compared to birch, for instance), and rapid growth [158,159,160]. 

A number of investigations of technology and performance of bamboo-based composites and their usability for wind turbines have been carried out. Holmes et al. [161] produced a novel bamboo-poplar epoxy laminate for blades formed by hot-pressing and carried out mechanical testing of the laminates (monotonic tensile and compressive stress-strain behavior and tension-tension fatigue life). Platts [162] presented strength test results for Brazilian Dendrocalamus Giganteus bamboo. He demonstrated the important effect of the moisture content on strength and stiffness and proposed to coat the bamboo with epoxy resin, to create a vapor barrier and preserve the dryness. Platts [163] also suggested to handle the variability of a natural material by making planks out of many parallel strips and then structures out of many parallel planks, creating averaging effect. Huang et al. [164] compared wood/epoxy laminate and bamboo/epoxy laminate, and demonstrated that the bamboo epoxy laminates have better mechanical properties. In [165], life cycle assessment of turbine blades from bamboo has been carried out. The authors showed that the properties of bamboo satisfy the requirement of materials for wind turbine blades, both from the materials quality viewpoint and from life cycle analysis viewpoint. 

With the increased emphasis on recyclability of structural materials, bamboo and bamboo-based composites attracted growing interest of several groups, especially in Asia [166]. Several Chinese companies developed and tested bamboo-based wind turbines [166]. Lianyungang Zhongfu Lianzhong Composites Group Co. produced 1.5 MW bamboo fiber composite wind turbine blade. Chinese company Miracle Zhufeng carried feasibility study for bamboo composite blades, with the availability of low cost materials sources. The company was, however, liquidated in 2012. 

Generally, bamboo-based composites were and are a promising direction for the sustainable wind turbine blades, with rather high technology readiness level, and a number of successful tests for small and medium turbines. 

### 6.3. Plant Based Composites and Bio-Based Polymers

Plant fiber-based composites are based on non-wood fibers, typically, grasses, in the form of yarns or chopped mats. According to [167] the plant fiber composites show better mechanical properties but more difficult processability than wood composites. 

Again, the use of the grass and fibers as reinforcements has a long history. These materials were used by ancient Egyptians as reinforcement for mud and clay bricks for the building of walls [168,169]. There is a large amount of research studies, reviews and proof of concepts in the area of plant-based composites for structural applications (see review, for instance, in [168,170]). The studies related to wind turbine blade applications are scarce, and deal mainly with small scale turbines. So, Shah et al. [171] investigated the manufacture and mechanical performance of full-scale 3.5-m composite rotor blades (for 11 kW turbines) from flax/polyester and E-glass/polyester composites. They observed that the flax blade satisfies the structural integrity requirements of blades under both “normal” and “worst case” operation. Kalagi et al. [172] reviewed mechanical properties of natural fiber composites, in view on their applicability in blades, and concluded that they can replace common glass fiber composites in the future. In [173], mechanical properties of hemp fibers in vinylester matrix were studied, and compared with glass fibers 

From the viewpoint of environmental sustainability, the natural fiber reinforced composites are not always preferable over common glass fiber reinforced composites. Birkved et al. [174] carried out life cycle assessment of sustainable fiber composites for the wind turbine blades, and concluded that the optimal material is not the 100% flax fiber composite, but rather 70% flax/30% carbon fibers (in terms of environmental sustainability performance) and 20% flax/80% carbon fibers (in term of costs). Corona et al. [175] studied the development of composites for blades by substitution of conventional fibre materials with bio-fibers, and compared different biofibers and hybrids with view on environmental sustainability. Using the life cycle impact assessment, the authors observed that the environmental sustainability of natural fiber based composite materials can be even lower, than that of the conventional materials, due to the high resin demand of natural fiber composites. 

Ideally, recyclable, sustainable composites should include not only bio-based fibers, but also bio-based, recyclable resins. Several companies and groups developed bio-based and/or biodegradable resins, among them Rilsan^®^, Altuglas^®^ Rnew and others from Arkema [176], FDCA and PEF from Avantium (not biodegradable, and also inert ester linkages), adhesive resins from Cambond (partly biobased since crosslinked with petroleum-based isocyanates) and so on [177]. Typically, they are based on polylactic acid/PLA, thermoplastic starch, polyhydroxyalkanoate/PHA, and cellulose. However, PLA degradation in water can limit the use of PLA based systems for durable and long-life structures. The Danish company Pond [178], developed high performance biobased biodegradable resin, which has a potential for wind turbine blades. 

Epoxidized linseed oil (ELO) was shown to be a promising substitution of the petroleum-based resin in the blades, thanks to similar hardness and improved toughness [179]. Schmidt and Reynaud and colleagues [140,141,142] developed high-performance bio-based epoxy/glass fiber composites, based on anhydride-cured epoxidized linseed oil formulation for maximum mechanical performance and vacuum-assisted resin transfer molding technology. Moller [138] manufactured glass fiber reinforced composites with ELO based matrix cured with various anhydride curatives and catalysts, and managed to prevent the void formation. 

A way to enhance mechanical and service properties of bio-based polymers is to use nanoengineered resins, matrix or fiber sizing, with nanocellulose secondary reinforcements. Hierarchical composites with secondary nanoreinforcements often demonstrate superior properties over conventional composites [180,181]. Nanoparticles strengthen polymer and slow down damage evolution. This was observed also for biocomposites [182,183]. Okubo and colleagues produced PLA/bamboo fiber/ microfilbrillated cellulose (MFCs) composites, with additions of just 1–2 wt.% of microfibrillated cellulose (MFC) [184]. The potential of NC reinforcement is very high; however, its efficiency depends on the quality of NC distribution and adhesion with the polymer. While the idea of hierarchical biocomposites was tested in laboratory, the idea has not yet been used to create new structural parts.

In [51,185], biobased polylactic acid (PLA) self-reinforced composites (combination of low melting PLA matrix with high stiffness, high melting PLA reinforcing fibers, both biodegradable) were developed. The material is easily recyclable thanks to one material structure (i.e., when both matrix and reinforcements are PLA), shows high impact resistance and higher failure strain. 

## 7. Discussion and Conclusions

The current, commonly accepted scenario of wind turbine blade life is 20 to 25 years of service, followed by incineration and landfill, recycling or reuse. However, according to [186], wind turbines (for instance, with blades from epoxy/glass fiber composites) can function without problems until they turn 50 years old. 

In view on the sustainability of technologies, the repair, reuse and refurbishment technologies show some advantages, as compared with recycling. Comparing the different end-of-life technologies, Joeman [79] obtained the climate change parameters: 2.02 × 10^5^ kg CO_2_ eq for using the blades for making pavements, 1.69 × 10^4^ kg CO_2_ eq for pyrolysis application, 3.12 × 10^3^ kg CO_2_ eq for refurbishing, 1000 kg CO_2_ eq for landfilling. Among other investigations, LCA studies were carried for one blade from a 2 MW wind turbine model, 6.5 tons, dismantled at a windfarm 300 km away from the processing facilities. 

According to [7,8], recycling and sustainable incineration of resources “hold limited economic and long-term sustainability benefits compared to the reuse, repair and remanufacturing of components”. According to [187], 71% of turbine emission comes from raw materials, 6% from manufacturing, 12% from operation and maintenance. The additional problem of blade recycling is the problem of handling all types of materials in the blade i.e. thermosets, fibers, PVC, PET, PU and balsa wood, coatings. In view of this, the extension of service time of already manufactured blades can be preferential, as compared to manufacturing new blades from new materials.

Repair and reuse are most mature technologies, among other available decommissioning scenarios. In [16], technology readiness levels of different technologies are listed, with relatively low TRL 3 for self-healing polymers, TRL 4 for reversible cross-linking of thermosets, TRL 6 for thermoplastic blades, and high TRL 9 for repair and reuse.

Thus, for now, the strategy of maintenance, repair, reuse and refurbishment has a number of advantages over other approaches (recycling, or new materials, or, on the other side, incineration) (which are in fact still not there). This means that the maintenance, repair and refurbishment technologies should be further developed, to reduce costs, and increase efficiency. This will provide an intermediate solution for coming years, until the new recycling technologies, and, on the other side, new generation of wind turbine blade materials come on the market. 

Many ideas of new generation of wind turbine blade materials were successfully tested, in laboratory, and in some cases in field. The rather common feature of these next generation solutions is that they in many cases can be well used for small or even medium size wind turbine blades, but their application for large wind turbines is still constrained, either due to inferior properties, or due to technological challenges. The bio-based composites in wind energy, e.g., natural fiber composites, wood based and bamboo composites, have been tested for small and medium blades, but still did not find application for large blades, due to the inferior mechanical properties of these materials, which are sufficiently lowed than the requirements toward the large wind blades. Sometimes, different performance criteria of the sustainability of wind turbine blade materials are considered separately, like recyclability, easy defect healing, durability, manufacturing from widely available and easily growing natural resources, and also environmental aspects of manufacturing. The materials development for wind turbine blades should include the aspects of sustainability, recyclability, improved lifetime, health monitoring, and maintenance easiness (reparability, health control, etc.).

## Data Availability

Data sharing not applicable. No new data were created or analyzed in this study.
